# Ras isoform-specific expression, chromatin accessibility, and signaling

**DOI:** 10.1007/s12551-021-00817-6

**Published:** 2021-07-31

**Authors:** Ruth Nussinov, Mingzhen Zhang, Ryan Maloney, Hyunbum Jang

**Affiliations:** 1 Computational Structural Biology Section Leidos Biomedical Research, Inc., Frederick National Laboratory for Cancer Research in the Laboratory of Cancer Immunometabolism National Cancer Institute, 1050 Boyles St, Frederick, MD 21702 USA; 2 Department of Human Molecular Genetics and Biochemistry, Sackler School of Medicine Tel Aviv University, 69978 Tel Aviv, Israel

**Keywords:** KRAS, HRAS, NRAS, K-RAS4A, K-RAS4B, Gene accessibility, Signaling, Inhibitor

## Abstract

The anchorage of Ras isoforms in the membrane and their nanocluster formations have been studied extensively, including their detailed interactions, sizes, preferred membrane environments, chemistry, and geometry. However, the staggering challenge of their epigenetics and chromatin accessibility in distinct cell states and types, which we propose is a major factor determining their specific expression, still awaits unraveling. Ras isoforms are distinguished by their C-terminal hypervariable region (HVR) which acts in intracellular transport, regulation, and membrane anchorage. Here, we review some isoform-specific activities at the plasma membrane from a structural dynamic standpoint. Inspired by physics and chemistry, we recognize that understanding functional specificity requires insight into how biomolecules can organize themselves in different cellular environments. Within this framework, we suggest that isoform-specific expression may largely be controlled by the chromatin density and physical compaction, which allow (or curb) access to “chromatinized DNA.” Genes are preferentially expressed in tissues: proteins expressed in pancreatic cells may not be equally expressed in lung cells. It is the rule—not an exception, and it can be at least partly understood in terms of chromatin organization and accessibility state. Genes are expressed when they can be sufficiently exposed to the transcription machinery, and they are less so when they are persistently buried in dense chromatin. Notably, chromatin accessibility can similarly determine expression of drug resistance genes.

## Introduction

Quantifying Ras isoform-specific expression in distinct tissues has been challenging. Here, we suggest that a key factor determining their cell-specific levels is their epigenetics and chromatin accessibility status. Chromatin accessibility relates to the local density level of proteins including histones, transcription factors, chromatin-modifying enzymes, and chromatin-remodeling complexes on the DNA. Their depletion at *cis*-regulatory elements commonly points to candidate genomic regions which may be available for transcription (Minnoye et al. 2021). Post-translational modifications (PTMs) of chromatin, such as DNA methylation, and histone methylation and acetylation, are dynamic, varying across cell states and types and correlating with chromatin accessibility and gene expression. The dynamic density levels make profiling of accessibility an extremely difficult task (Ashwin et al. 2019; Barth et al. 2020; Wachsmuth et al. 2016). An added complexity is the interpretation of the data as it relates to enhancer–promoter proximity and functional transcription factor binding (Minnoye et al. 2021). As we discuss below, these complexities combine with additional ones underscoring the challenge of quantitative studies of Ras isoform-specific expression.

**Fig. 1:**
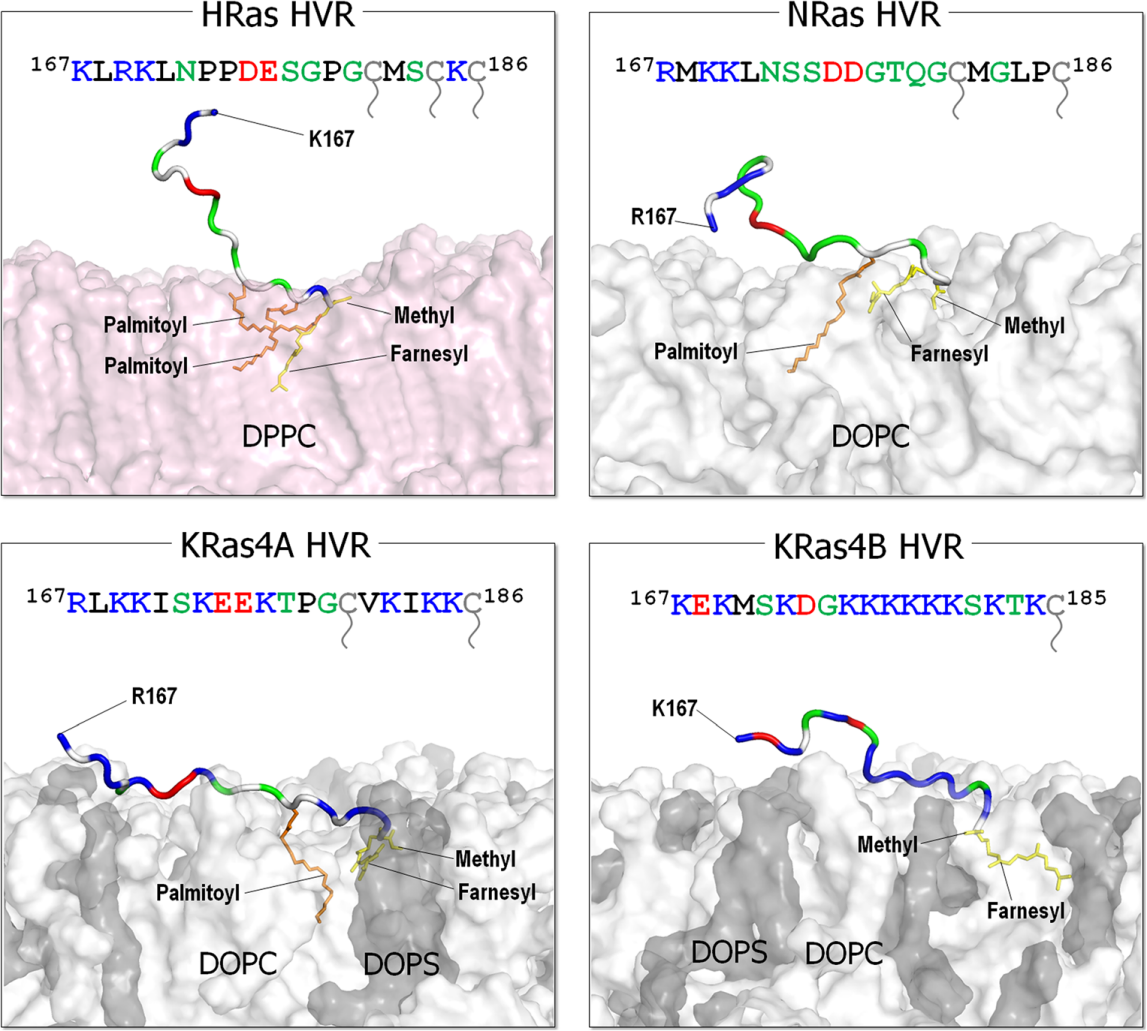
The sequences of C-terminal hypervariable regions (HVRs, residues 167–185/186) of the Ras isoforms and the modeled HVR structures with the post-translational modifications (PTMs) representing their preferred membrane interactions. The modeled HRas and NRas HVR peptides preferentially bind the zwitterionic DPPC or DOPC bilayers in the gel or liquid phases, while the KRas HVR peptides anchor to the anionic DOPC/DOPS bilayer in the liquid phase. In the HVR sequence, basic residues (positively charged) are colored in blue, acidic residues (negatively charged) are colored in red, hydrophobic residues are colored in black, and polar and glycine residues are colored in green. The prenylated Cys residues are colored in gray with a tail mark. In the HVR cartoon, the same colors are used, except the hydrophobic residues colored in white. The farnesyl and palmitoyl groups are shown as yellow and orange sticks, respectively. In the surface representation of lipid bilayer, pink, white, and gray surfaces denote DPPC, DOPC, and DOPS, respectively

Here, we describe isoform-specific activities and review experimental observations from a structural dynamic standpoint. This conformational perspective of isoform-specific activities mimics nature: biomolecules are not static sculptures. Molecular behavior, which dominates the structure–function paradigm, is shaped by biomolecules which are always switching between a variety of structures with varying energies, with the most populated being those which are energetically most favored. This dynamic (Kumar et al. 2000; Tsai et al. 1999) behavior can be described by the free energy landscape (Frauenfelder et al. 1991). The populations of the conformational species are influenced by multiple factors, including sequence alterations, which dictate specific interactions. Thus, with different sequences and chemistries (charge, hydrophobicity) and distinct combinations of hydrophobic PTMs in the hypervariable C-terminal tails (Fig. 1), the isoforms present different favored interactions with membrane lipids and protein partners.

Our views are influenced by concepts from physics and chemistry. We believe that molecular fluctuations are harnessed for life (Nussinov 2016; Wei et al. 2016) and that biomolecules must be described statistically, not statically. Understanding functional specificity requires insight into how biomolecules can organize themselves and their assemblies in different cellular environments, including molecular concentrations (e.g., of scaffolding proteins, as was shown for galectin-1 (Blazevits et al. 2016)). It is also manifested by the distinct segregations into nanoclusters, and the isoform dynamics between the substructures where they congregate and their movements in the plasma membrane into—and out of—the membrane domains (Nussinov et al. 2019b).

## Background information on Ras

### Ras isoforms, structure, and biology

Ras proteins control cell proliferation pathways, cell growth and division (Lavoie et al. 2020; Nussinov et al. 2020b; Smith et al. 2020). Three *RAS* genes lead to four proteins, HRas, NRas, and splice variants KRas4A and KRas4B. The incidence of mutated *RAS* genes differs among human cancers: *KRAS* is the most highly mutated isoform (85%), followed by *NRAS* (11%), and *HRAS* (4%). They predominate in different cancer types and the distributions of the mutations differ as well (Cox et al. 2014; Hobbs et al. 2016; Li et al. 2018a; Prior et al. 2012). Sequence comparisons indicate that the catalytic domains of the isoforms (residues 1–166) are almost identical. Binding to exchange factors exchanges GDP by GTP activating the Ras isoforms; binding to GTPase-activating proteins (GAPs) deactivates them (Tsai et al. 2015; Vigil et al. 2010). When activated, Ras proteins bind and activate their effectors, initiating their respective signaling pathways, primarily mitogen-activated protein kinase (MAPK) via Raf/MEK/ERK (MEK, mitogen-activated protein kinase kinase; ERK, extracellular signal regulated kinase) and PI3K/AKT/mTOR (PI3K, phosphatidylinositol 3-kinase; AKT, protein kinase B; mTOR, mechanistic target of rapamycin) phosphorylation cascades. Both signaling pathways feed into the cell cycle, together leading to cell proliferation (Nussinov et al. 2017).

**Fig. 2:**
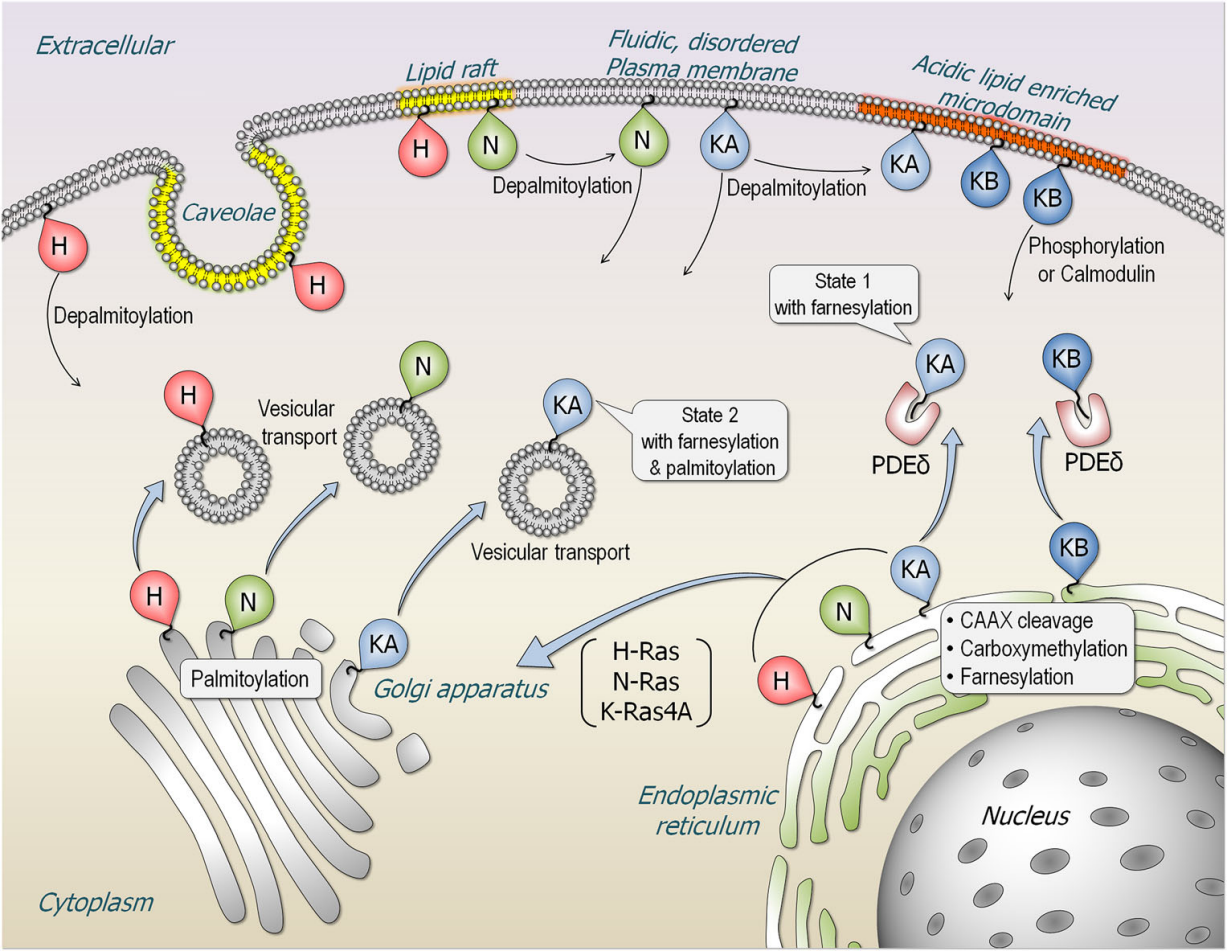
Subcellular localization of Ras proteins. Ras isoforms, HRas, NRas, and two splice variants KRas4A and KRas4B terminate in a CAAX motif are cleaved, carboxymethylated, and farnesylated in the endoplasmic reticulum (ER). HRas, NRas, and KRas4A are further palmitoylated in the Golgi apparatus, while KRas4B traffics to the plasma membrane shuttled by phosphodiesterase-δ (PDEδ). KRas4A in state 1 (only farnesylated) can also traffic to the plasma membrane via a similar mechanism as in KRas4B. After palmitoylation, HRas, NRas, and KRas4A in state 2 (with both farnesyl and palmitoyl modifications) are translocated to the plasma membrane via vesicular transport. HRas favors localization in the ordered caveolae and lipid rafts, and fluidic disordered (non-raft) regions of the plasma membrane. NRas can localize to cholesterol-rich lipid raft and non-raft regions. Depalmitoylation removes HRas and NRas from the plasma membrane. KRas4A in state 2 can localize to the non-raft region, while both KRas4A in state 1 and KRas4B highly localize to the acidic lipid enriched membrane microdomains. Phosphorylation of Ser181 or calmodulin extracts KRas4B from the plasma membrane

Over 150 experimentally determined structures of the catalytic domain have been deposited in the structural database. In contrast, the 22–23 residue C-terminal tail which constitutes the hypervariable region (HVR) is disordered, precluding crystallization. However, its populated conformations have been sampled by NMR and molecular dynamics (MD) simulations (Chavan et al. 2015a). Membrane anchorage, executed by the tails, is required for Ras activation, with the tails’ hydrophobic PTMs docking at favored membrane environments (Fig. 1). The HVRs are farnesylated, proteolyzed, and carboxyl methylated. In addition, NRas, HRas, and KRas4A cysteines are palmitoylated. These PTMs stabilize the anchorage. Lacking a palmitoyl, KRas4B attachment to negatively charged membranes is assisted by a lysine polybasic stretch (^175^KKKKKK^180^). The covalent palmitoylation thioester modification linkages are reversible; the farnesyl thioether linkages are not. The HVR of KRas4B can also be phosphorylated. The introduction of the large, negatively charged phosphoryl group dissociates KRas4B from the negatively charged plasma membrane (Bivona et al. 2006; Jang et al. 2015). The HVR of KRas4A resembles that of KRas4B: it is also highly positively charged, albeit not to the extent of KRas4B (Nussinov et al. 2016). Whereas the lysines are almost continuous in KRas4B, they are not in KRas4A and there are fewer of them (Tsai et al. 2015). KRas4A’s pattern of PTMs varies as well and lies in-between NRas and KRas4B. Like NRas, KRas4A can have farnesyl and palmitoyl; however, with the palmitoyl thioester linkage being reversible, it may be hydrolyzed, resulting in KRas4A retaining only its farnesyl. This can explain KRas4A’s acting as NRas (when the tail is farnesylated and palmitoylated) and as KRas4B (when the palmitoyl is hydrolyzed), thus NRas- and KRas4B-like cell transformation patterns (Tsai et al. 2015). Like the C181S NRas, a C180S mutation in KRas4A does not stop it from shuttling to the plasma membrane. However, in the absence of palmitoylation and diminished polybasic region as compared to KRas4B, KRas4A retains lower affinity for the plasma membrane (Chakrabarti et al. 2016; Muratcioglu et al. 2017; Nussinov et al. 2016). The HVR sequence and palmitoylation status also govern the isoforms’ preferred plasma membranes (Eisenberg et al. 2011) (Fig. 2). The positively charged KRas HVR, but not HRas or NRas, strongly favors acidic disordered membranes (Fig. 1), making membrane composition an important consideration in isoform-specific signaling (Chavan et al. 2015b; Nussinov et al. 2018a). Isoform-specific HVR activities also include binding to membrane shuttling factors as in the case of phosphodiesterase-δ (PDEδ) which shuttles KRas4B (Dharmaiah et al. 2016; Klein et al. 2019; Kuchler et al. 2018; Muratcioglu et al. 2017). NMR data and MD simulations of GDP-bound KRas4B suggest that the HVR obstructs access to the active site (Banerjee et al. 2016; Jang et al. 2016a), which apparently is not exhibited by other isoforms likely due to the absence of a sufficiently strong positive charge. Ras isoforms are also differentially ubiquitylated, which may affect their membrane attachment status (Ahearn et al. 2018; Dohlman and Campbell 2019; Hobbs et al. 2016; Sasaki et al. 2011). Activation-related effects include monoubiquitylation at Lys147, which increases the levels of KRas4B-GTP due to impaired GAP binding (Baker et al. 2013a; Sasaki et al. 2011), and HRas ubiquitylation at Lys117, which promotes GDP-GTP exchange (Abe et al. 2020; Baker et al. 2013b).

### Earlier discussions on Ras isoforms

Several comprehensive reviews of Ras isoforms have been published in the last few years. These broadly discussed their different biochemical and biological (many cancer-related) roles, localization, sublocalization, and more. To avoid re-reviewing the topics which were covered, we refer the reader to these recent excellent publications. These include subcellular localization and considering exploiting the membrane in therapeutics (Kattan and Hancock 2020; Zhou et al. 2018), mutational analysis and isoform signaling (Li et al. 2018a; Munoz-Maldonado et al. 2019; Prior et al. 2020; Randic et al. 2021), subcellular localization and tumor growth (Garcia-Ibanez et al. 2020), Ras–ERK signaling (Zaballos et al. 2019) and MAPK inhibition (Heppner and Eck 2021; Ullah et al. 2021), isoform-specific differences in the effector binding regions (Nakhaeizadeh et al. 2016), and the recent contributions from the Mark Philips lab on KRas4A reversible palmitoylation and colocalization (Amendola et al. 2019) and on membrane association/colocalization (Zhou et al. 2020). Isoform signaling specificity at the membrane (Nussinov et al. 2018a), KRas mobility in the membrane (Nussinov et al. 2019b) and nanoclustering (Nussinov et al. 2019a) were also reviewed as well as genetic aspects of KRas signaling networks (Jinesh et al. 2018). Thus, below, we only briefly touch on some of these aspects. Instead, we provide new views which we hope will help guide future research.

### Ras isoforms segregate into nanoclusters which favor distinct membrane composition: family members with similar tail chemistry can join

**Fig. 3:**
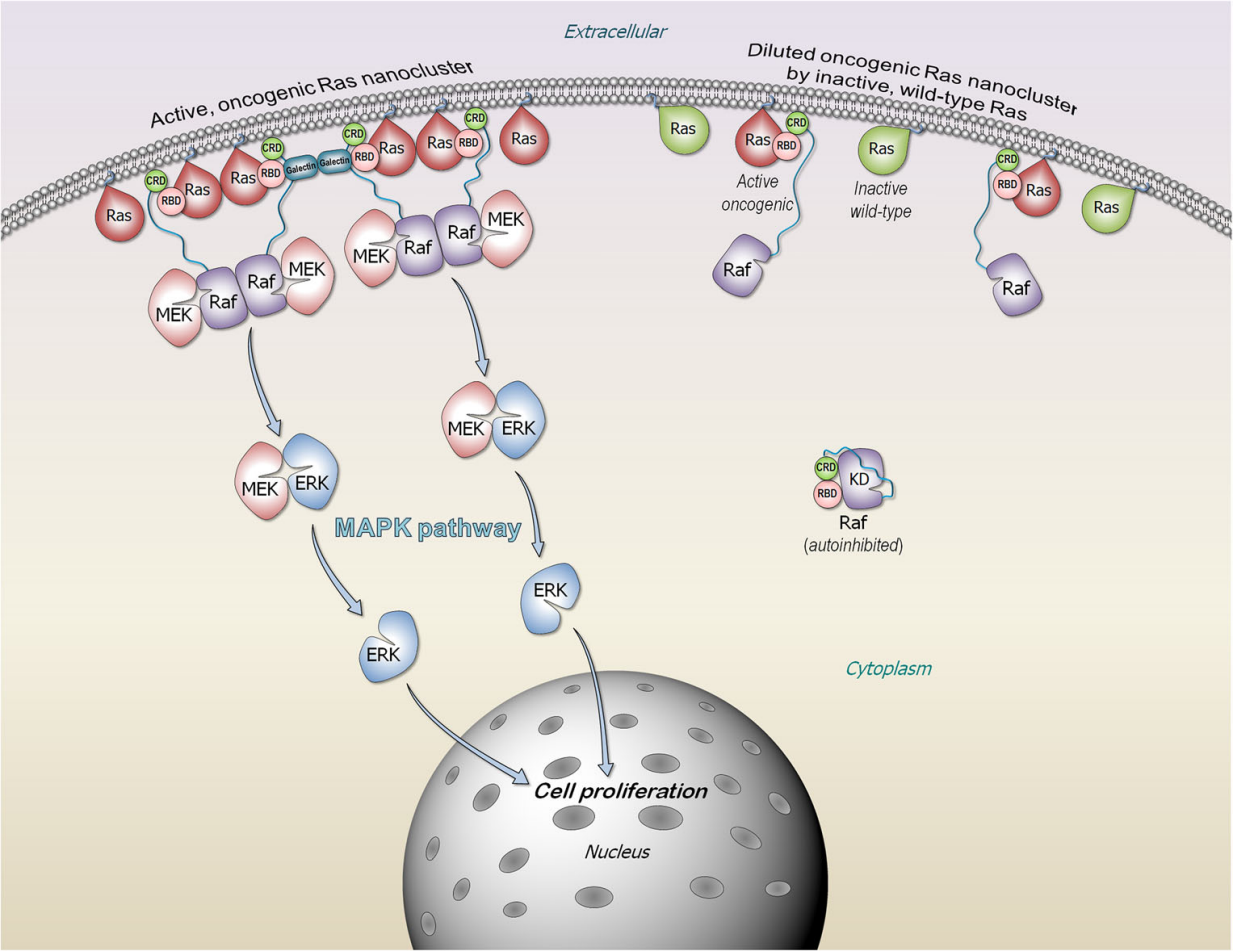
Active, oncogenic Ras forms nanocluster at the membrane microdomain that can serve as a signaling platform. Ras nanocluster recruits Raf kinases to the membrane microdomain, promoting Raf kinase domain dimerization, activation, leading to signaling via the MAPK (Raf/MEK/ERK) phosphorylation pathway. Monomeric Raf is mainly autoinhibited in the cytosol. Ras releases the autoinhibition via the high-affinity interaction of Ras with Raf RBD. Inactive, wild-type Ras dilutes the oncogenic Ras nanoclusters. The wild type normally exists in the cell in its GDP-bound form. Its intrinsic orientation on the membrane surface occludes its effector binding site, which is buried by the membrane, abrogating effector binding and suppressing Raf’s activation. The cartoon was inspired by previous publication of MAPK suppression by Rap1 (Nussinov et al. 2020a)

GTP-bound Ras forms nanoclusters in the membrane. Nanoclustering is required for Ras signaling via the MAPK, but not PI3Kα/AKT/mTOR pathways (Bandaru et al. 2019; Boriack-Sjodin et al. 1998; Cherfils and Zeghouf 2013; Hancock et al. 1989; Jang et al. 2016a; Jang et al. 2016b; Muratcioglu et al. 2017; Nussinov et al. 2018a; Nussinov et al. 2019b; Schmick et al. 2014; Zhou and Hancock 2015). The reason for this distinction is that for MAPK signaling, Raf kinase domains need to be activated. This requires their side-to-side (homo- or hetero-) dimerization (Freeman et al. 2013a; Freeman et al. 2013b; Jambrina et al. 2016; Lavoie et al. 2018; Rezaei Adariani et al. 2018; Tsai and Nussinov 2018; Udell et al. 2011; Varga et al. 2017; Verkhivker 2016; Yuan et al. 2018), which is not the case for PI3Kα (Nussinov et al. 2018a; Nussinov et al. 2019a). The two kinase domains are contributed by two Raf molecules, with the Ras binding domain (RBD) of each Raf binding to the catalytic domain of an active Ras molecule (Fig. 3). This requires that Ras molecules either be in direct contact (Ras dimers) (Jang et al. 2020; Muratcioglu et al. 2020; Nan et al. 2015) or in spatial vicinity which can be achieved in sufficiently populated nanoclusters. Membrane anchored nanoclusters increase the probability of such favorable proximity (Nussinov et al. 2019a), and galectin dimers were proposed to scaffold Raf-effectors to further promote Ras nanoclustering (Blazevits et al. 2016). In this model, at high concentrations, galectin dimerizes in the cytoplasm and binds to two Raf’s RBDs which are Ras-bound. The galectin–Raf complexes cooperatively nucleate Ras nanoclustering, thereby promoting dimerization of Raf’s kinase domains. Computational modeling supported by experimental data suggested that cooperativity between KRas4B dimerization and Raf-1 (C-Raf) RBD–Ras binding may also emerge though the engagement of Raf-1’s cysteine-rich domain (CRD) at the membrane (Jang et al. 2020). In this model, Raf-1’s RBD-CRD can cooperatively support stable KRas4B dimers/nanoclusters. The varied chemistry of the HVRs, thus membrane preferences, favors distinct isoform nanoclusters. However, recently KRas4B nanoclusters were shown to be co-inhibited also by other isoforms and notably, by a subset of prenylated small GTPase family members, confirming the importance of dimer/co-cluster formation on cell membranes (Li et al. 2020b), in line with an earlier hypothesis making such prediction (Nussinov et al. 2020a).

A few years ago, wild-type HRas was observed to suppress HRas driven cancer (Spandidos et al. 1990). This was followed by the observation that wild-type KRas can inhibit lung cancer (Diaz et al. 2002) and that wild-type NRas can also suppress its mutant as potently as can KRas (Zhang et al. 2001). However, the data (To et al. 2013; Xu et al. 2013; Zhou et al. 2016) as to how would wild-type Ras suppress the oncogenicity of its mutant was not understood (Kong et al. 2016; Qiu et al. 2011; Staffas et al. 2015) despite its significant implications to Ras drug discovery (Zhou et al. 2016). Nanoclustering can resolve these apparent perplexing observations. In the absence of an external cue, wild-type Ras spends most of its life in the inactive state. “Diluting” the population of active, oncogenic Ras by the inactive wild type would lower the probability of spatially nearby active Ras molecules (Fig. 3), thus Raf dimerization, activation, and MAPK signaling. This effect is particularly large in cells with high KRas populations. Segregated nanoclustering also resolves the next question of why the wild-type state of one isoform type cannot suppress the oncogenic mutant of another (Matallanas et al. 2011; Xu et al. 2013). The apparent observed suppression of MAPK by Rap1A small GTPase, whose HVR resembles that of KRas4B, might also be explained along similar lines (Nussinov et al. 2020a).

#### Ras isoforms display distinct favored interactions with Raf isoforms

Ras/Raf/MEK/ERK is a major Ras signaling pathway involving protein kinases phosphorylation cascade (Lavoie and Therrien 2015). The sequences, structures and functions of Raf isoforms, A-Raf, B-Raf, and Raf-1 are mostly similar, and they share conserved regions (CRs); CR1, CR2, and CR3 (Shaw et al. 2014; Yaeger and Corcoran 2019). CR1 at the N-terminal, contains the RBD and the CRD. In active Raf, RBD interacts with Ras, and CRD binds to the membrane (Garcia-Gomez et al. 2018; Ghosh et al. 1994; Li et al. 2018b; Terrell and Morrison 2019; Travers et al. 2018) (Fig. 3). Inhibition of Raf can allosterically promote its paradoxical activation (Hatzivassiliou et al. 2010; Heidorn et al. 2010; Poulikakos et al. 2010) via heterodimeric interactions between the kinase domains of Raf isoforms (Jin et al. 2017; Pfister et al. 2008; Ritt et al. 2010). Dimerization of the kinase domains is required for full activation (Durrant and Morrison 2018; Hu et al. 2013; Lavoie et al. 2013; Rajakulendran et al. 2009). Under normal circumstances, B-Raf/Raf-1 heterodimers predominate (Freeman et al. 2013a).

Wild-type Raf is mostly in the “closed” autoinhibited state, where the CRD and RBD shield the kinase domain dimerization surface (Nussinov et al. 2020c), with the 14-3-3 proteins stabilizing this organization (Kondo et al. 2019; Park et al. 2019). Dephosphorylation of pSer365 in B-Raf (pSer259 in Raf-1) and release of RBD-CRD following a growth activation signal at the membrane relieve the autoinhibition. The high affinity binding of Raf’s RBD to active Ras, coupled with pSer365 dephosphorylation which destabilizes the 14-3-3 interaction, shifts the Raf ensemble toward the open state (Zhang et al. 2021). This exposes the kinase domain dimerization surface enabling full activation (Fetics et al. 2015; Hatzivassiliou et al. 2010; Lu et al. 2016; Nussinov et al. 2015; Nussinov et al. 2019a). Cryo-EM structures (Kondo et al. 2019; Park et al. 2019) of the Raf kinase domain, RBD and CRD complexed with dimeric 14-3-3 validate the organization of the assembly and the paradoxical activation of Raf (Kondo et al. 2019).

**Fig. 4:**
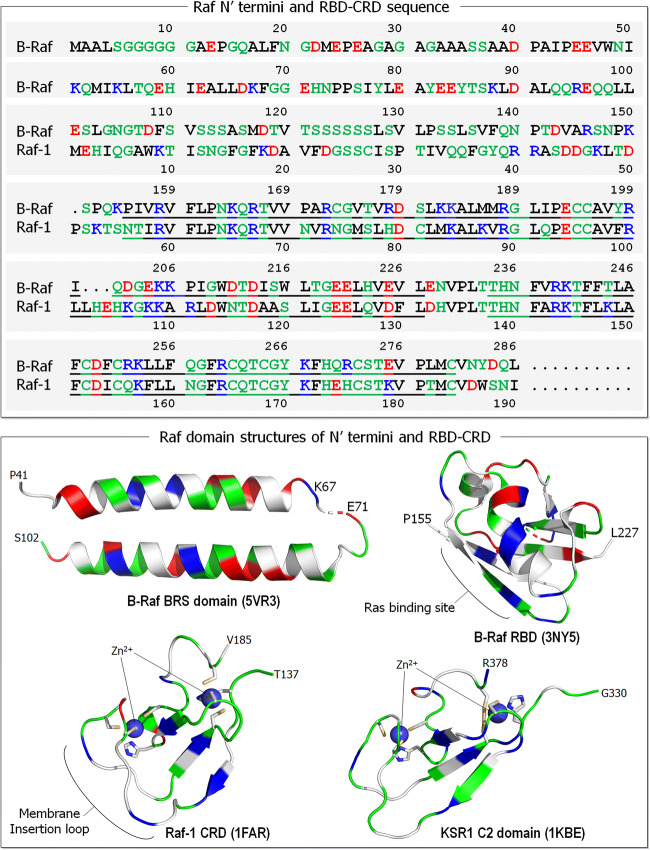
Sequences of the N′ termini and RBD-CRD of Raf isoforms, B-Raf and Raf-1 (upper panel). In the sequences, basic residues (positively charged) are colored in blue, acidic residues (negatively charged) are colored in red, hydrophobic residues are colored in black, and polar and glycine residues are colored in green. The B-Raf N′ terminus is longer (154 residues) than that of Raf-1 (54 residues). Underlines denote the RBD and CRD residues. A collection of domain structures for Raf N-terminal region are shown in the lower panel. Crystal structures of B-Raf-specific (BRS) domain (PDB: 5VR3) and B-Raf RBD (PDB: 3NY5). Solution structures of Raf-1 CRD (PDB: 1FAR) and kinase suppressor of Ras 1 (KSR1) C2 domain (PDB: 1KBE). In the cartoon, the same colors are used as in the sequence, except the hydrophobic residues colored in white. In Raf-1 CRD, sticks highlight the Cys152, Cys155, His173, and Cys176 residues coordinated with the first Zn^2+^, and the His139, Cys165, Cys168, and Cys184 residues coordinated with the second Zn^2+^. Similarly, for KSR1 C2 domain His334, Cys359, Cys362, and Cys377 coordinated with the first Zn^2+^, and Cys346, Cys349, His367, and Cys370 coordinated with the second Zn^﻿2+^

Recent experiments probed the preferred interactions of Ras isoforms with Raf isoforms, by shuffling their N′ termini, which precede the RBD-CRD. The N′ termini vary significantly among the Raf isoforms (Fig. 4), whereas it is long (154 residues) and negatively charged in B-Raf, it is more neutral in Raf-1 (55 residues) and A-Raf (18 residues). The experiments indicated that the N′ termini of Raf isoforms selectively bind the HVRs of Ras isoforms (Terrell et al. 2019). Replacing the Raf-1 terminus with B-Raf’s reduced the HRas–Raf-1 binding but had no significant effect on KRas–Raf-1 interaction. As to Ras HVRs, Raf-1 has high affinity to all Ras isoforms. However, the lysine-rich polybasic region of KRas4B is important to B-Raf selectivity due to its acidic N′ terminal region, resulting in the high-affinity KRas4B–B-Raf RBD interaction. Taken together, Raf-1 binds all Ras proteins; however, B-Raf favors KRas4B, with the sequences responsible for these preferences residing in the N-terminal (for B-Raf) and the HVR at the C-terminal for KRas4B. Additionally, Raf-1 is important for the HRas interaction, with a fairly neutral HVR.

Even though these observations provide critical information, understanding them is marred by lack of structural data. This reflects the disordered nature of the N′ termini of Raf, especially the long B-Raf sequence. It is also unclear how the HVR concomitantly interacts with the membrane and Raf’s termini. With structural data only for a fraction of the sequence, it is challenging to reliably model it.

#### Isoform-specific localization

Two-decades ago, Alan Wolfman raised the apt question of “How is it that similar proteins carry out different jobs in the cell?” (Wolfman 2001); his answer was “Location, Location, Location.” He proceeded to survey literature reports and concluded that different functions may stem from the distinct subcellular localizations to which the isoforms are directed by their HVRs and patterns of prenylation. Nonetheless, even if Ras isoforms are localized to unique sets of cellular structures, the number of Ras isoform-specific interaction sites is limited, which underscores the importance of understanding why they apparently signal through distinct pathways. This may imply that a Ras isoform-dependent oncogenic phenotype necessitates (i) cooperation of additional cellular Ras proteins, (ii) a steady-state pool of their complexes, and (iii) availability of other proteins in the pathway in the cancer cell. Notably, Ras sublocalization at the plasma membrane is highly dynamic and can be altered depending on the GDP/GTP load or the palmitoylation status (Agudo-Ibanez et al. 2015). Electron microscopy indicated that HRas is mainly in the endoplasmic reticulum and Golgi of acinar cells; KRas in the membrane of ductal cells. Overall, Ras isoforms were observed to have distinct and separate cellular and subcellular distributions that likely persist even in transformed cells (Kocher et al. 2005).

## Differential Ras isoform expression

### RAS isoforms are preferentially expressed in different cancer types

The question of why the oncogenic *RAS* isoforms are preferentially expressed in different cancer types has been baffling (Der 2014; Hobbs et al. 2016), and several hypotheses have been proposed to address it (e.g., Der 2014; Haigis et al. 2008; Lampson et al. 2013; Rauen 2013; Schuhmacher et al. 2008; more below). Among these, here we reason that the patterns of isoform expression in different cells can relate to the accessibility of the gene’s regulatory regions. The density of chromatin in regulatory regions of highly expressed isoforms could be lower than of those with lower expression. Temporal expression profiles of different isoforms across developmental stages identified “isoform switching” of the predominant isoform ( >60% of all isoforms of the given gene at the given stage) (Li et al. 2020a). The chromatin density of specific genes differs between differentiated cells as compared to cells during embryogenesis. The local chromatin density can also vary among the differentiated cells, making the regulatory regions of some genes more accessible to the transcription machinery than others. This, along with the epigenetic features, control gene expression (Klemm et al. 2019), including in our case here wild-type and mutant Ras isoforms (Nussinov et al. 2021), thus signaling.

This picture becomes even more complex when Raf’s expression and mutational patterns are considered (Desideri et al. 2015). Mutations in B-Raf, but not in A-Raf and Raf-1, are common in human cancers, with Raf-1 mutations at under 1% (Imielinski et al. 2014). Mutations were observed in pancreatic and lung adenocarcinoma and colorectal cancer, where KRas4B is commonly involved. It is overexpressed in bladder cancer, hepatocellular carcinoma, squamous cell carcinoma, and lung adenocarcinoma (Maurer et al. 2011) and is MEK independent (Blasco et al. 2011; Karreth et al. 2011).

### Quantitative data on isoform-specific expression are limited

Isoform-specific expression has been probed, as well as its relation to signaling (Newlaczyl 2016) and prognosis (Yang and Kim 2018). KRas4B was confirmed as the most highly expressed isoform and KRas4A as the most dynamically regulated. Quantification of Ras isoform expression during development by real-time polymerase chain reaction (PCR) in mouse tissues indicated a relative contribution of KRas4B   > >   NRas ≥ KRas4A   >   HRas to total Ras expression (Newlaczyl et al. 2017), where KRas4B is about 60–99% of all Ras transcripts. Recent data have also suggested that spliced variants are translationally significant (Raso 2020). This may reflect the dependence of multiple factors, including cell type, tissue heterogeneity, timing, sparsity of data, defining flexible statistical frameworks for complex differential patterns in gene expression, assigning a reference, errors and missing data, and from our standpoint as we discuss here, key factors are measuring chromatin accessibility (Buenrostro et al. 2015; Cusanovich et al. 2015) and epigenetics, such as DNA methylation (Karemaker and Vermeulen 2018) and more (Lahnemann et al. 2020).

HRas, KRas, and NRas have specific context-dependent functions (Hobbs et al. 2016; Nussinov et al. 2018a; Nussinov et al. 2020b), and mutations (Li et al. 2018a; Munoz-Maldonado et al. 2019). They also display cancer type specific incidence: KRas in pancreatic, lung, and colorectal carcinomas, NRas in cutaneous melanoma (Cox et al. 2014), and HRas in head and neck and bladder squamous cell carcinomas. Several theories have been put forward to explain the cell (tissue)-specificity. Among them (Der 2014), (i) isoform specificity reflects the level of expression. Yet, the significantly higher incidence of *KRAS* mutations as compared to *NRAS* (Haigis et al. 2008) and *HRAS* (Schuhmacher et al. 2008) in colorectal carcinoma was offered as questioning this explanation. Another explanation (ii) relates to possible differential potencies in promoting cancers across tissues (Haigis et al. 2008; Russo et al. 2014). (iii) An alternative explanation offered differential DNA repair as a consequence of Ras isoform-specific activating mutations (Ise et al. 2000). This suggested that *KRAS* regulatory elements are responsible for tissue specificity, rather than the Ras protein (Chin et al. 1997; To et al. 2008). Still other explanations suggested (iv) that isoform translational efficiency encoded by codon usage could be the origin of the isoform specificity (Lampson et al. 2013) and finally, as analysis of The Cancer Genome Atlas indicated (v), tumor *RAS* gene expression levels are influenced by the mutational status of *RAS* genes and of upstream and downstream Ras pathway genes (Stephens et al. 2017).

**Fig. 5:**
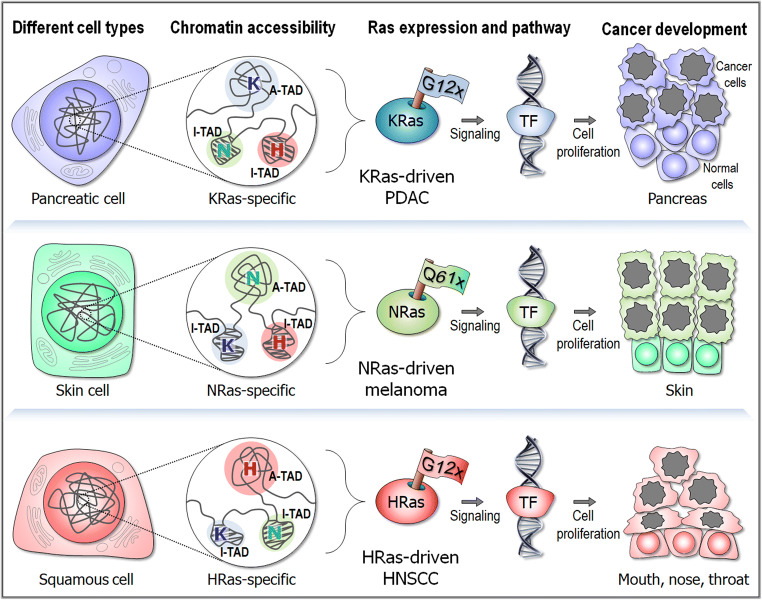
Dynamic chromatin accessibility and cell-specific signaling pathways of Ras isoforms. KRas G12x mutations dominantly drive pancreatic ductal adenocarcinoma (PDAC), while NRas Q61x mutations are often found in melanoma. HRas mutations frequently occur in head and neck squamous cell carcinoma (HNSCC). Ras isoform expression in different cell types can relate to chromatin accessibility states of specific topologically associating domains (TAD) (Szabo et al. 2019). In the cell nucleus, the disordered chain represents the chromatin, a chromosome in interphase. Examples of different chromatin accessibility for *RAS* isoform-specific TAD conformations are highlighted in the circles. In the highlighted chromatin, A-TAD and I-TAD denote active and inactive TADs, respectively. K, N, and H indicate isoform-specific genes in the chromatin. Oncogenic Ras stimulates cancer signaling toward the transcription factor (TF) bound DNA that regulates the transcription of genes to cell proliferation. The phenotype of the production is cancer development

Furthering the differential potencies of mutants in promoting cancers across tissues (Haigis et al. 2008; Russo et al. 2014), we propose a role for chromatin accessibility (Fig. 5). The genome of all cells is identical. However, not all genes are equally expressed during the developmental lineage and across tissue microenvironments. One major reason is the status of the chromatin density in gene regulatory regions. Regulatory regions can be buried in compact dense chromatin or be in low-density regions. In low density regions, the local chromatin conformation is controlled by nucleosome dynamics. These regions can become available to the transcription machinery upon a relatively minor conformational change (Nussinov et al. 2020b). There is a continuum of accessibility across the genome (Klemm et al. 2019), stretching from genes expressed only in embryonic cells, to those in differentiated cells, as in the case of Ras isoforms. Accessibility reflects the cell’s epigenetic landscape (Haigis et al. 2019; Sack et al. 2018) and relates to the density of proteins interacting directly (mostly histones) or indirectly with the DNA and their fractional residence times. Accessibility is a major factor determining gene expression, thus protein availability and consequently, pathways that consist of interactions of these proteins. Protein availability differs among tissues: protein levels in pancreatic cells where KRas expression is abundant may differ from those in skin cells, where NRas is (Nussinov et al. 2020b). Taken together, KRas and NRas can be differentially expressed in specific cell types because their chromatin accessibility status differ (Brubaker et al. 2019). This could clarify why the heightened abundance of the same active mutant, e.g., KRas^G12D^, would differ in pancreatic cancer and melanoma skin cancer. On the other hand, the differential outcome of NRas^Q61R^ *versus* NRas^G12D^ in melanoma may stem from the differential mutation strengths, which depends on the activation mechanisms (Burd et al. 2014). Exploring the respective mechanisms would be of interest and could aid in development of isoform- and mutant-specific inhibitors.

### Chromatin accessibility and genome organization are cell-type and cell-state specific

To fit into the limited nuclei space, act in regulation and guard genome integrity, chromatin is compacted. Compactness has been assessed by several methods, including a quantitative fluctuation-based assay (Hinde et al. 2012) and FRET; however, the low resolution does not permit correlation of the in vivo signals with specific higher order chromatin organization (Lleres et al. 2009). Quantitative super-resolution microscopy assay (Dultz et al. 2018) and algorithms for the quantification of chromatin condensation from microscopic data have been developed, but to date their applications have been limited (Sosnik et al. 2017). Chromatin accessibility reflects changes in the local density, or compaction (Magana-Acosta and Valadez-Graham 2020). Dynamic changes in chromatin landscapes are associated with cell differentiation during embryogenesis and dedifferentiation in pluripotent stem cell (iPSC) in cancers. Among the factors defining chromatin states are the composition and post-translational modifications (PTMs) of the nucleosomes, concentration and interaction of transcription factors, and chromatin remodelers (Klemm et al. 2019). The mechanisms controlling accessible chromatin regions include competition between transcription factors and histones, chromatin remodeling in *cis* through proximal linker histone displacement, and in *trans* through accessible, distal regulatory elements, binding of the pioneer transcription factors to nucleosomal DNA and more (Klemm et al. 2019). Landscapes vary in different tissues and cell types.

Most chromatin conformation capture experiments focused on intrachromosomal interactions. Recent observations reveal that the patterns of interchromosomal interactions are tissue-specific, differing between the heart and liver (Chapski et al. 2018). The experiments (Nothjunge et al. 2017; Rosa-Garrido et al. 2017) show preferential localization of genes in 3D in the nuclei of the organs in which the genes are transcribed. Comparisons of liver and cardiac chromatin structures identify widespread differences in compartmentalization, with these not fully correlating with the organ transcriptional states. Localization of genes within organ-specific chromatin scaffolds relate to cell type but can reflect stress conditions as well. Genome structures indicate that the promoter to transposable element loops differ between the organs, pointing to cell type specific organization of the epigenome. Interchromosomal interactions were enriched in genes associated with the function of that cell type, localizing nearby (Cremer and Cremer 2001) in “transcription factories” (Papantonis and Cook 2013). In the heart, 66.7% of cardiac-specific genes are in the center of one compartment (marked compartment A), while 66.1% of liver-specific genes are toward the periphery in compartment B. The locations of the Ras isoforms on the human chromosomes also differ (Pellicer 2011; Rajasekharan and Raman 2013): *HRAS* gene is localized to the short arm (p) of chromosome 11 at position 5, the *KRAS* gene is located on the p arm of chromosome 12 at position 12.1, and the *NRAS* gene is on chromosome 1 at position 13.2. Even though these are positions along the linear chromosome sequence organization, and to date data about their 3D locations are unavailable, the distinct locations of isoforms suggest distinct organization and expression patterns.

Advancements in super-resolution imaging (e.g., Nir et al. 2018) coupled with measurements of mRNA expression in distinct cell types and states can test whether the patterns of Ras isoform expression are associated with chromatin accessibility (Nussinov et al. 2021; Nussinov et al. 2020b).

## Ras isoforms, their functions, and cell signaling

### Differential isoform signaling

A key question is how a specific pathway can be selected when the affinities of the effectors for each Ras isoform do not show appreciable differences (Sieburth et al. 1998; Wolfman 2001). That is, how can highly similar proteins carry out different actions in the cell? Possible explanations to this conundrum include (i) cell type-specific expression levels of the Ras isoforms, and of all nodes (proteins) in the respective pathway. For the signal to propagate, the levels of expression of these nodes need to be sufficiently high. (ii) Observations for over two decades (reviewed in, e.g., Castellano and Santos 2011; Garcia-Ibanez et al. 2020; Hobbs et al. 2016; Kattan and Hancock 2020; Mo et al. 2018) suggest that isoform functions may emerge from the subcellular localization favored by their HVRs (Wolfman 2001) (Fig. 1). Isoform-specific functions of Ras can be at least partly explained by localization (Fig. 2). For example, NRas and HRas, but not KRas, are expressed on the Golgi as well as the plasma membranes and it was recently reported that this localization inhibits malignant transformation (Casar et al. 2018). KRas4A but not KRas4B directly regulates hexokinase 1 by virtue of its unique localization on the outer mitochondrial membrane driven by depalmitoylation (Amendola et al. 2019). Compartmentalized signaling based on HVR-driven subcellular localization has been established in model organisms (Onken et al. 2006) and finally, a stark example of differential signaling from distinct subcellular locations is in T cell thymic selection (Daniels et al. 2006). (iii) Mutational potency is often isoform tissue specific (Munoz-Maldonado et al. 2019). The strong KRas4B^G12D^ driver interferes with GTP hydrolysis. It occurs broadly, but especially in pancreatic cancers. In contrast, the less frequent, weaker KRas4B^A146T^ drives cancer by promoting GDP by GTP exchange. It has been observed in colorectal and hematopoietic cancers, but not in pancreatic adenocarcinomas where it is not sufficiently powerful for cell transformation (Bera et al. 2019; Poulin et al. 2019).

### Major considerations in signaling outputs in distinct tumors

Thus, taken together, signaling outputs in *distinct tumors* reflect several major components. These include first, the expression levels which depend on chromatin accessibility in the respective cell type at that time window (Fig. 5). The local density of chromatin at the regulatory region of the gene and nearby in *cis* has to be low to permit binding of the transcription machinery and high expression rates. Indeed, even very high expression level on its own can promote cancer. Second, expression of other proteins in the respective pathway should not be low for the signal to propagate. Third, the mutations should be potent. When considering different mutations in the *same cell/tumor*, heightened abundance of activated Ras species depends on the expression level of that gene and the potency of the mutation. The NRas^Q61R^ mutations versus KRas^G12D^ in melanoma cell line provide an example (Burd et al. 2014). As clinical data have shown, the number of activated KRas^G12D^ molecules in pancreatic cancer is extremely high.

### Calmodulin interacts selectively with oncogenic KRas4B

With a negatively charged linker and hydrophobic pockets, calmodulin (CaM) interacts with KRas4B (Abraham et al. 2009; Chavan et al. 2013; Jang et al. 2017; Jang et al. 2019; Villalonga et al. 2001; Wu et al. 2011), and likely also with KRas4A, but not with the HRas and NRas isoforms (Nussinov et al. 2016). The high affinity charge-charge interaction coupled with the farnesyl nestling in CaM’s hydrophobic pocket, shifts the ensembles toward this energetically favored state, extracting KRas molecules from the membrane (Fivaz and Meyer 2005; Sidhu et al. 2003; Sperlich et al. 2016). While CaM’s interaction appears KRas4B GTP-dependent (Abraham et al. 2009; Chavan et al. 2013; Villalonga et al. 2001; Wu et al. 2011), it can also involve the GDP-bound state (Agamasu et al. 2019; Fivaz and Meyer 2005; Sidhu et al. 2003; Sperlich et al. 2016). This can be understood in terms of the availability of the HVR for the interaction (Jang et al. 2019). In solution, the HVR in the GDP-bound state interacts with the catalytic domain, populating an autoinhibited state (Chavan et al. 2015a; Lu et al. 2015). However, the interaction is unstable which is why it has not been captured in the crystal, suggesting a minor GDP-bound KRas4B population with the HVR available for CaM interaction (Jang et al. 2016a; Nussinov et al. 2018b). At the membrane, likely being sandwiched between the catalytic domain and the bilayer surface the autoinhibited state can persist (Jang et al. 2016a).

CaM’s binding to mutant KRas4B is vastly important to understand. Two possible reasons have been advanced to explain its role: (i) CaM–KRas4B-specific binding reduces the number of available free CaM molecules for Ca^2+^-dependent protein kinase II activation (Wang et al. 2015); (ii) phosphorylated CaM and mutant KRas4B bind PI3Kα and activate it (Joyal et al. 1997; Wang et al. 2018; Zhang et al. 2017; Zhang et al. 2018). Together, mutant KRas4B and CaM can stimulate the PI3Kα/AKT pathway even in its absence of an incoming receptor tyrosine kinase growth signal. CaM’s fundamental significance in KRas-driven adenocarcinoma made it a prime drug discovery target.

## Conclusions

Even though there are some sequence differences in the catalytic domains, the distinction among Ras isoforms rests mainly in their HVR membrane-binding segments (Fig. 1). This distinction underscores the significance of the attachment to membrane domains in determining isoform functions, cellular sublocalization and shuttling vehicles to get them there (Fig. 2). The chemical uniqueness of the HVRs stemming from the variable amino acid sequences and the combination of prenyl modifications, with the consequent separation into mostly homogeneous nanoclusters, emphasizes their specific roles under normal conditions and the resulting mutational distributions observed in cancer. As recent work elegantly demonstrated (Terrell et al. 2019), Ras isoforms interact differentially with Raf isoforms, with Raf-1 binding all mutant Ras proteins with high affinity, whereas B-Raf exhibiting a strong preference for mutant KRas. It is thus quite likely that Ras isoforms also differentially interact with other Ras effectors, such as PI3Kα (Thevathasan et al. 2013). Even though differential KRas and HRas regulation by galectin isoforms was also observed (Elad-Sfadia et al. 2004; Shalom-Feuerstein et al. 2005), more recently the interaction was proposed to be mediated via Raf’s RBD (Blazevits et al. 2016), thereby cooperatively scaffold Ras nanoclusters, which would increase dimerization of Raf’s kinase domains and activation.

All proteins are encoded and can be expressed by all cells (Kosti et al. 2016). However, genes are preferentially expressed in tissues (Farahbod and Pavlidis 2019; Honore 2020): proteins expressed in pancreatic cells may not be equally expressed in lung cells. This holds for isoforms of Ras and other proteins, including receptor tyrosine kinases and lipid kinases. It is the rule—not an exception, and it can be at least partly understood in terms of chromatin organization and incoming signaling cues (Fig. 5) and mRNA levels (Lorch and Kornberg 2017; Rolicka et al. 2020). Genes are expressed when they can be sufficiently exposed for the transcription machinery to trigger remodeling of the chromatin conformation, and they are less so when they are persistently buried in dense chromatin (Magana-Acosta and Valadez-Graham 2020). Chromatin remodeling takes place in cancer (Arildsen et al. 2017; Lafon-Hughes et al. 2008; Morgan and Shilatifard 2015). Cell type and cell state epigenetic organizations are key factors determining gene expression status, and the expressed proteins are wired in the cellular protein-protein interaction network. Super high-resolution electron microscopy and computational prediction methodologies are rapidly advancing, and they are being applied to cell-specific cancer genomes. This raises hope that *RAS* isoform-specific gene scale topologically associating domains (TAD) will be identified not only in specific tissues (Szabo et al. 2019), but also at different cell-transformation states. Such detailed maps could help forecast gene expression and alternative signaling pathways in drug resistance (Nussinov et al. 2021; Nussinov et al. 2020b). The anchorage of Ras isoforms in the membrane and their nanoclustering have been studied extensively, including their detailed interactions, sizes, and preferred membrane environments, chemistry and geometry (Lee et al. 2019). However, the challenge of their epigenetics and its linkage to rewired networks in distinct cell states and types is still waiting to be unraveled. The landscape of accessibility changes dynamically in response to external and developmental cues (Klemm et al. 2019). But its tissue-specific footprints may help in deciphering the impending pathways in drug resistance.

To date, pharmacology has successfully targeted KRas4B^G12C^ (Zeng et al. 2020; Zhang et al. 2020). Ras isoform-specific pharmacology at the membrane has been deliberated. However, considering membrane fluidity, the common presence of phosphatidylserine, and the non-uniqueness of the PTMs, toxicity is a challenge. Whereas pharmacological innovations are compelling, reliably forecasting future developments is formidable, making the harnessing of the signaling pathways appear more tractable venues.
